# Increased resolution of African swine fever virus genome patterns based on profile HMMs of protein domains

**DOI:** 10.1093/ve/veaa044

**Published:** 2020-06-19

**Authors:** Charles Masembe, My V T Phan, David L Robertson, Matthew Cotten

**Affiliations:** College of Natural Sciences, Makerere University, Makerere Hill Road, P. O Box 7062 Kampala, Uganda; Viral Genomics, Wellcome Trust Sanger Institute, Hinxton, Cambridge CB10 1SA, UK; Department of Viroscience, Erasmus Medical Centre, Dr. Molewaterplein 40, 3015 GD Rotterdam, The Netherlands; MRC University of Glasgow Centre for Virus Research, 464 Bearsden Rd, Glasgow G61 1QH, UK; Viral Genomics, Wellcome Trust Sanger Institute, Hinxton, Cambridge CB10 1SA, UK; MRC University of Glasgow Centre for Virus Research, 464 Bearsden Rd, Glasgow G61 1QH, UK; MRC/UVRI & LSHTM Uganda Research Unit, P.O. Box 49, Plot 51–59 Nakiwogo Road, Entebbe, Uganda

**Keywords:** large and complex genome classification, virus catastrophic for global food production

## Abstract

African swine fever virus (ASFV), belonging to the *Asfarviridae* family, was originally described in Africa almost 100 years ago and is now spreading uncontrolled across Europe and Asia and threatening to destroy the domestic pork industry. Neither effective antiviral drugs nor protective vaccines are currently available. Efforts to understand the basis for viral pathogenicity and the development of attenuated potential vaccine strains are complicated by the large and complex nature of the ASFV genome. We report here a novel alignment-free method of documenting viral diversity based on profile hidden Markov model domains on a genome scale. The method can be used to infer genomic relationships independent of genome alignments and also reveal ASFV genome sequence differences that determine the presence and characteristics of functional protein domains in the virus. We show that the method can quickly identify differences and shared patterns between virulent and attenuated ASFV strains and will be a useful tool for developing much-needed vaccines and antiviral agents to help control this virus. The tool is rapid to run and easy to implement, readily available as a simple Docker image.

## 1. Introduction

African swine fever virus (ASFV), belonging to the *Asfarviridae* family, was first described in Kenya nearly 100 years ago (Eustace Montgomery 1921). The virus is endemic in most sub-Saharan African countries where it naturally infects warthogs and bush pigs and is frequently transmitted via soft ticks. In sub-Saharan Africa, infections of warthogs and bush pigs have a typically mild disease outcome. In domestic swine or wild boars, ASFV infections can result in a more serious disease with much greater mortality: between 90 per cent and 100 per cent. Of great concern for animal welfare and the food industry, ASFV infections are responsible for increasing swine mortality in several parts of the world (Pikalo et al. 2019). Outside of Africa, the virus has previously been reported in Portugal, and in Haiti in sporadic outbreaks, probably as a result of imports from West Africa (Bastos et al. 2003; Phologane, Bastos, and Penrith 2005). Since the virus’s first appearance in Georgia in 2007, the virus has spread to wild boar populations in Europe (reviewed in Cwynar, Stojkov, and Wlazlak 2019), with currently 3,608 cases reported and a further 1,413 cases in swine as of 1 June 2019. A disturbingly high prevalence of ASFV has been found in Chinese dried pig blood used as porcine feed additives with all 21 tested samples testing positive by polymerase chain reaction (PCR) in a recent study and a full ASFV genome sequence assembled (Wen et al. 2019). Furthermore, ASFV sequences have been identified in Chinese pork imported into Korea (Kim et al. 2019). These recent European and Asian incursions and outbreaks involve p72-Genotype II (GII) ASFV and appear not to involve the soft tick stage as originally observed in some parts in Africa. At the time of writing, neither antiviral drugs/agents nor an effective vaccine is available to stop the epidemic.

The ASFV virion is enveloped and spherical or pleomorphic in shape with a diameter of 175–215 nm. The virus has a linear, dsDNA genome of 170–195 kb with complementary terminal sequences. The ASFV genome encodes >150 open reading frames (ORFs; Dixon et al. 2013). In addition to known viral structural and replication proteins, there are a large number of ORFs with undefined functions. These include the multigene families (MGFs) that show frequent duplication, deletion, or inversion across the virus family (Dixon et al. 2013). Multiple examples of attenuated ASFV variants encoding changes in their MGF content indicate that these genes have a role in ASFV virulence (Aguero et al. 1990; Almendral et al. 1990; Gonzalez et al. 1990; Rodriguez et al. 1994; Zsak et al. 2001; Afonso et al. 2004; Burrage et al. 2004; Netherton, Rouiller, and Wileman 2004; Golding et al. 2016). However, the complexity of the MGFs and the nature of their sequence changes in ASFV evolution make it difficult to accurately ascribe specific changes in the ASFV genome to changes in phenotype. A simplified tool for monitoring these potentially functional changes would benefit the field and may aid in making a safe attenuated vaccine strain as well as to guide efforts to develop antiviral therapies.

The p72 gene (∼1,950 bp) is frequently used for PCR diagnosis of ASFV (Atuhaire et al. 2013). Additional genes used for the diagnosis include the central variable region of pB602L gene and p54 protein (encoded by E183L gene, an antigenic structural protein involved in viral entry). Currently, there are twenty-four ASFV genotypes described based on p72 sequences (Mulumba‐Mfumu et al. 2019), with the two most recent genotypes found in Ethiopia (Achenbach et al. 2017) and Mozambique (Quembo et al. 2018). There have been efforts to classify ASFV strains, including using three ORFs (Gallardo et al. 2009; Michaud, Randriamparany, and Albina 2013; Rock 2017; Alkhamis et al. 2018), the p72 gene (Onzere et al. 2018), and the pB602L gene (Sanna et al. 2017). In general, these methods have been limited to small portions of the ASFV genome (i.e. <1% of the genome size), which are not likely to capture the full evolutionary history of the virus. Important drivers for this research activity are efforts to understand the pathology of the virus infection, the components of a protective immune response, and, a priority for vaccine development, the generation of attenuated but still immunogenic virus strains that may be used for vaccination. Altogether, better understanding of ASFV biology will help prevent and control the transmission of this virus across continents.

We have been developing the use of encoded protein domains as a classification tool for viral genomic sequence data, for example, applied to *Coronaviridae* genome sequences (Phan et al. 2018). Instead of using differences in nucleotide or protein sequences to identify possible changes across sets of evolutionary-related viral genomes, employing the domain classification would inform, not only the genome changes but also the potential functional alterations of the virus genomes. All protein domains are well described in the Pfam collection, available at https://pfam.xfam.org. Novel instances of a domain and its relative distance to a reference domain can be rapidly identified in query sequences using the software HMMER-3 (Eddy 2011). HMMER package can be used to perform similarity searches using profile(s) against a protein sequence database (hmmsearch program) or, alternatively, using protein sequence(s) against a protein profile database (hmmscan). By using Pfam as the database of profile hidden Markov models (HMMs), it is possible to identify functionally defined protein domains that are encoded by a viral genome. A matrix of these domain scores can then be used to compare and cluster sets of ASFV genomes in an approach that is similar to a sequence-based phylogenetic analysis. We applied this domain comparison method to explore ASFV genome diversity and evolutionary relationships, to provide some functional clues for differences in viral genomes, and to help identify viral elements associated with attenuation, virulence, or transmissibility.

## 2. Materials and methods

Collection of the ASFV genomes. All ASFV full genomes were retrieved from GenBank (5 April 2019) using the query: txid137992[Organism] AND 170000[SLEN]:200000[SLEN] yielding forty-eight complete genomes. Two genomes were identical: genome MK333180 and genome MK33318, the latter having been derived from dried blood products, only MK333180 was retained for a final set of forty-seven genomes. The GenBank entries and original literature were searched for country, date, and original host (tick, warthog, wild boar, or domestic pig) as well as any indication of virulence derived from the original literature. A summary of the 47 genomes used for the analysis is provided in Supplementary Table S1.

### 2.1 Pfam-A domain content

The Pfam domains encoded by ASFV genomes were identified using the hmmsearch function of HMMER-3.2.1 (Eddy 2011), searching against the most recent Pfam database (Pfam 32.0, September 2018, 17929 entries; Finn et al. 2016; El-Gebali et al. 2019). For each genome in the collection, all ORFs were translated from both reading strands (using biopython). Proteins ≥75 amino acids were used as queries against profile HMMs of the Pfam database. A domain hit was retained if the domain independent E-value (domain_i-Evalue) was ≤0.0001. Details of each domain instance were gathered, including the position in the query genome, the length, the domain_i-Evalue, and the bit-score.

### 2.2 Custom profile HMMs for the MGFs

All ASFV encoded MGF protein coding sequences were retrieved from GenBank as follows. An initial query to the NCBI nucleotide database was made to retrieve complete or nearly complete ASFV genomes (txid137992[Organism] AND 170000[SLEN]:200000[SLEN] NOT patent). From the ‘Send to’ menu, the option ‘all coding sequences’ was selected and these entries were retrieved to a fasta file. MGF entries were selected from the complete ASFV coding sequence file by sorting for the presence of the term “MGF” in the coding sequence ID with a simple python script. This yielded a set of 660 MGF entries.

When screened for Pfam content, 127 of the 660 protein coding sequences failed to return a domain hit (at a lenient domain_i-Evalue cutoff of 0.01). These were classified in GenBank as MGF_100 (thirty-eight entries), MGF_110 (nine entries), MGF_300 (thirty-nine entries), and MGF_360 (forty-one entries). To increase resolution for ASFV genome comparisons, profile HMMs were prepared for these proteins as follows. The 660 MGF ORFs were clustered using Usearch (Edgar 2010) at an aa fraction sequence identity of 0.75. Initially clustering pilots were performed at identities of 0.95, 0.90, 0.85, 0.80. 0.75, 0.70, and 0.65 (the lowest ID cutoff recommended for Usearch clustering). The 0.75 clustering gave the best separation of the coding regions into groups that corresponded to the GenBank annotation. In general, clustering followed the annotation, however several MGFs were further divided into subfamilies at this identity cutoff resulting in a set of forty-five MGF subfamilies. Each MGF subfamily was aligned using Mafft (Katoh and Standley 2013), and a profile HMM built using hmmbuild (Eddy 2011).These custom profile HMMs were used in combination with the identified Pfam profile HMMs (see Section 3).

The computational tools for performing this analysis are openly available as a platform independent Docker image of the tool and instructions for installing and using the tool have been made available (see Data availability section and Readme document in the Supplementary Data). The Docker image contains the Unix, python, biopython SciKit, and HMMER-3 modules needed to run the classification, and the set of 511 HMMs (469 from Pfam plus 45 custom profileHMMs from MGF families) which were used to classify ASFV genomes. Outputs from the classification tool are a clustermap, showing the relationship between the genomes and a comma-separated value (CSV) table listing all domains identified in each genome, their position, length, and coding strand in the genome and a flag indication high (≥0.03) or low variance (<0.03). This CSV table is useful for investigators wishing to explore the identified domains further or to investigate differences between genomes.

### 2.3 “UK” domain analysis

The ASFV encoded UK protein coding sequence was originally described by Zsak et al. (1998) and was analyzed both because it has been associated with virulence and because we want to demonstrate the link between domain bit-scores and protein identity. UK protein coding sequences were retrieved from the GenBank entry NC_001659 for the BA71V strain and used in an online BLAST search (MEGABLAST default settings) to identify closely related sequences. Using the download menu, all hits (thirty-nine entries, 1 October 2019) were retrieved to a fasta file, the UK domain coding sequence from the NC_001659 genome was added, and the set was translated into protein sequences using Geneious, aligned in Mafft (Katoh and Standley 2013), and Geneious was used to calculate pairwise aa differences and to visualize protein changes across the alignment. The Pfam domain content of the UK protein coding sequence set was determined as described above, identifying only the UK domain at a domain_i-Evalue cutoff of ≤0.0001. The domain bit-scores were collected for the set and compared with the pairwise aa differences (see Supplementary Fig. S1).

The forty-seven ASFV full genome sequences available in GenBank were aligned using Mafft (Katoh and Standley 2013) and the resulting alignment manually checked in AliView (Larsson 2014). Maximum likelihood (ML) phylogenetic tree of the p72 gene was constructed in RAxML (Stamatakis 2014) under the GTRGAMMA model of substitutions and bootstrapped for 100 pseudoreplicates. The tree was mid-point rooted for clarity and branches were drawn to the scale of nucleotide substitutions per site, and bootstrap values ≥75 per cent are shown on the internal nodes.

## 3. Results

**Figure 1.**
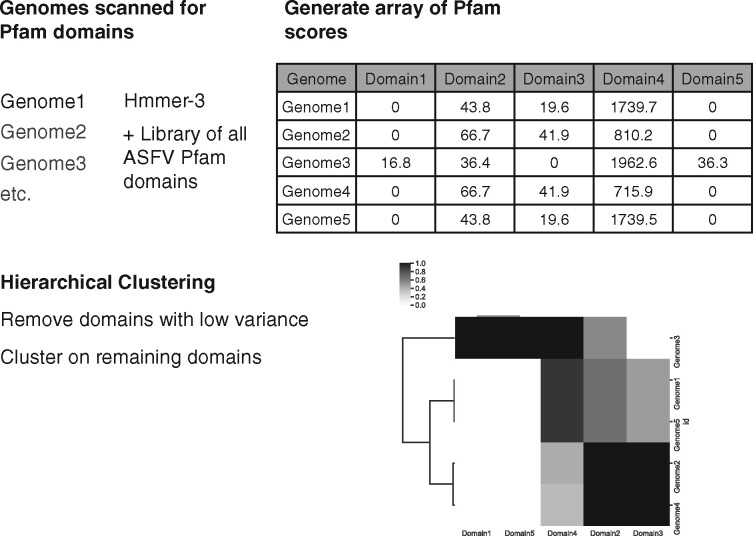
The process of genome clustering with profile HMMs. Each full ASFV genome was scanned for Pfam and MGF domain content (step 1), the domain scores were collected, built into a matrix, and normalized to fraction of highest score in the set (step 2). Domains with low variance across the entire set were removed, and hierarchical clustering of the genomes was performed using the high variance domains (step 3).

Initially, we identified all regions from the forty-seven ASFV genomes coding for proteins positive for profile HMMs of the Pfam collection. Using a domain_i-Evalue cutoff of 0.0001 (a measure of the number of expected hits that should be found by chance, given a database of the same size), eighty-two domains were identified at least once per genome in the set of forty-seven genomes, and seventeen domains were found twice or more per genome in the set indicating repeat occurrences in some genomes (see Supplementary Table S2). The domain content and their scores (from Pfam plus custom MGF domains) were then used to examine patterns of the forty-seven ASFV genomes in GenBank in the following manner. Briefly, for each genome, a total score for each domain was generated by summing the individual domain scores (taking into account multiple instances of the same domain). For each domain column in the matrix, the scores were normalized by dividing each value by the maximum value; domains that showed >0.03 variance in their score across the set of forty-seven genomes were retained and used for hierarchical clustering. A schematic presentation of the process is shown in Fig. 1.

### 3.1 Domain variability

As an illustration of the domain classification approach, we examined the UK gene’s ORF encoding a ninety-six aa protein expressed early in ASFV infection (Zsak et al. 1998). Although the protein is nonessential for growth in porcine macrophage cell cultures, deletion of the UK coding region reduces the virulence of ASFV in domestic pigs (Zsak et al. 1998). A set of ASFV UK coding regions was retrieved from GenBank, an alignment of the proteins set is shown in Supplementary Fig. S1A, revealing twenty-two aa differences between the most divergent forms of the protein. Following the HMMER-3 search of the UK ORFs, the Pfam domain score (bit-score) for the UK domain varies across the set with a bit-score value of 227.7 for perfect match. In support of the use of this metric, there is a highly significant negative correlation between Pfam domain score with the pairwise aa distance (Supplementary Fig. S1B). Of note, the Pfam UK domain entry was constructed using the ASFV reference strain NC_001659 UK protein as a model and the HMMER-3 score is correlated with the differences of query domains from this early ASFV sequence. Thus, an HMMER-3 search can be used both to find members of a domain family in a query genome and to provide a quantitative score (bit-score) of the distance of the query domain from the model domain.

### 3.2 Documenting Pfam content of ASFV

We identified all profile HMM domains from the Pfam collection which were encoded in a set of forty-seven ASFV genomes. Using a domain_i-Evalue cutoff of 0.0001 (a measure of the probability of finding the domain by chance), eighty-two domains were identified at least once in the set of forty-seven genomes, and seventeen domains were found twice or more in the set indicating repeat occurrences in some genomes (see Supplementary Table S2). As described above, the domain content and their scores (from Pfam plus custom MGF domains) were then used to examine patterns of the forty-seven ASFV genomes in GenBank.

**Figure 2.**
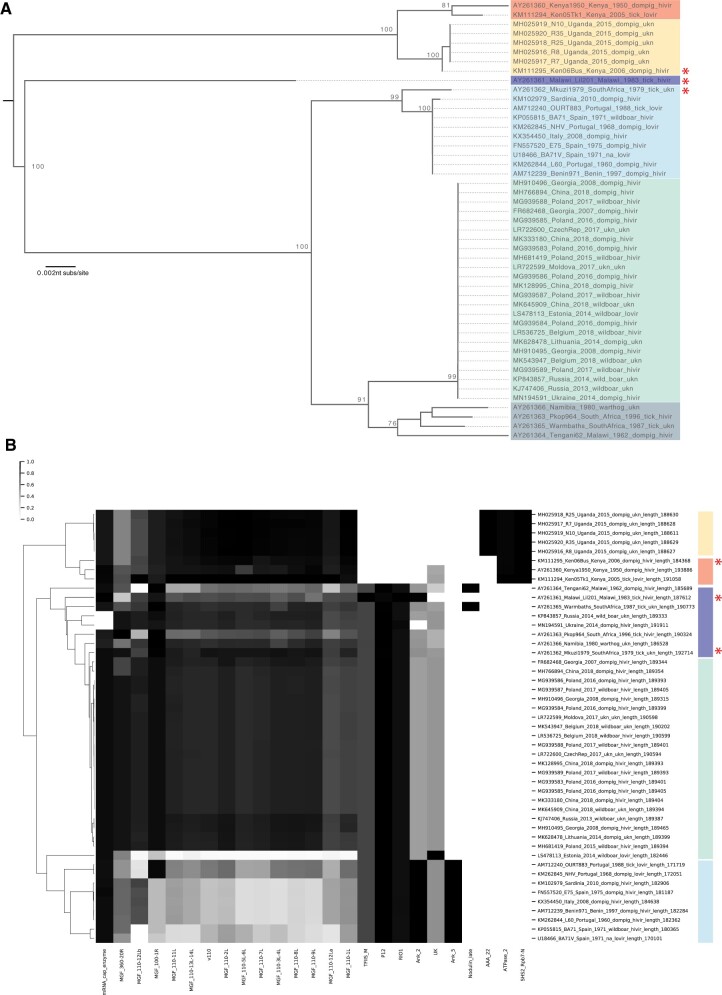
A: The p72 ML phylogenetic tree. The coding sequences of p72 gene from the forty-seven ASFV genomes available in GenBank were aligned in AliView. An ML tree was inferred using RAxML under GTRGAMMA model of substitutions with 100 bootstraps (see Section 2 for further details). The tree was mid-point rooted for clarity and branches were drawn to the scale of nucleotide substitutions per site (indicated in nucleotide substitutions/site), and bootstrap values ≥75 per cent are indicated. Genotypes are indicated by colored boxes, with the GII in green. B: The domain clustermap classification of forty-seven ASFV genomes. The forty-seven ASFV genomes were examined by their Pfam content (see Section 2). The bit-scores for all domains identified with domain_i-E-value ≤0.0001 were collected for each domain, a matrix was prepared and subjected to hierarchical clustering (see Section 2) based on domains whose normalized values showed ≥0.03 variance. In both panels, the genotypes are indicated with colored boxes. Genome IDs shown on node labels (A) and *Y* axis (B) include GenBank accession number, strain name, country, date, host, virulence, and length in nucleotides. For both panels, genomes with incongruent placement between the two methods are highlighted with a red asterisk.

The forty-seven full ASFV genomes were ordered by hierarchical clustering based on the Pfam + MGF domain scores and compared with a p72 ML tree with the major genotypes in each analysis indicated by colored boxes (Fig. 2). In validation of our approach, the domain-clustering (Fig. 2B) group genomes in nearly the same pattern as p72 ML tree topology (Fig. 2A), which is a current standard practice to genotype ASFV strains. Differences include the phylogenetic position of older genomes and those genomes obtained from tick samples. Of note, the GII viruses that are spreading globally clustered into a monophyletic group on the p72 ML tree (green shaded, Fig. 2A). Interestingly, the domain clustering showed that the Estonian genome (GenBank LS478113, identified from a wild boar in 2014; Zani et al. 2018) possesses a large 14-kb deletion, lacking functional domains MGF_110 1 L-12L compared with other GII ASFV viruses (Fig. 2B). Additionally, within the GII ASFV viruses, strains FR682468 and MH766894 show changes in the DUF4509 domain (associated with MGF_360 genes). In addition to diversity in the MGF domains, there is diversity (with variance ≥0.03) in the eleven domains (AAA_22, Ank_2, Ank_5, ATPase_2, mRNA_cap_enzyme, Nodulin_late, P12, RIO1, SHS2_Rpb7-N, TFIIS_M, and UK) observed across different genotypes. None of these domain absence/presence are revealed from a p72 ML tree (Fig. 2A) that is typically used to genotype these viruses.

### 3.3 Domains associated with MGFs

Five MGFs have been defined (MGF 100, 110, 300, 360, and 505/530) with the naming based on the mean number of amino acids in the gene product.

**Figure 3.**
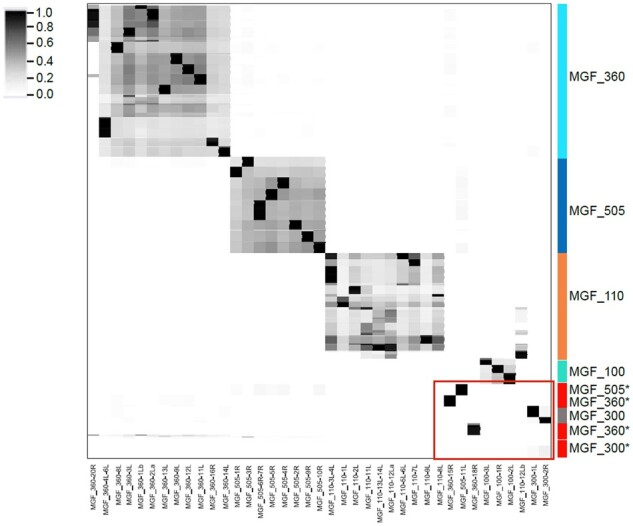
Hierarchical clustering of all available ASFV MGF protein sequences. All available ASFV MGF proteins (*N* = 660) were retrieved from GenBank, clustered at an amino acid fractional identity 0.85 and a profile HMM was prepared from each of the forty-five alignments (ASFV_HMM45) using HMMER3 (Eddy 2011). The same set of 660 proteins were then examined for ASFV_HMM45 content at a domain_i-Evalue threshold of 0.0001, bit-scores were collected and used to prepare a matrix describing the set of proteins. The matrix was then subjected to hierarchical clustering and a clustermap prepared. Each column represents one of the forty-five profile HMMs, each row represents an MGF protein. Major clusters are indicated to the right, unconventional domains that do not cluster with other members bearing the same GenBank MGF family annotation are marked in the red box.

All annotated ORFs from forty-seven complete genome entries in GenBank were collected (660 total entries, MGF_100: 38; MGF_110: 148; MGF_300: 46; MGF_360: 267; MGF_505: 160 entries) and examined for Pfam domains. Three MGFs consistently encoded at least one domain (i.e., all members of that MGFs were found to encode a particular domain). These were MGF_110: domain v110, MGF_360: domain ASFV_360, MGF_505: domain DUF249. To capture the diversity in these MGFs, we prepared individual profile HMMs from a comprehensive set of MGF ORFs. Briefly, we grouped each MGF protein by aa sequence identity, identified forty-five MGF subfamilies and then constructed custom profile HMMs for each of these (see Section 2). We then analyzed the clustering pattern of all MGF ORFs based on their custom profile HMMs (Fig. 3). Most MGFs clustered within their annotated family, evidenced by the rectangle of shared score similarities surrounding the large clusters of MGF_100 and MGF-110, MGF_360, MGF_505 (Fig. 3). However, a subset of ten MGFs appeared different from the main MGF group bearing their name (Fig. 3, red boxes, IDs with asterisks). For example, several ORFs annotated as MGF_505-11L have <0.85 aa sequence identity (fractional identity, Edgar 2010) with other MGF_505 family member and their domain scores cluster them to a unique sector of the graph (Fig. 3, red box). There is a similar pattern for MGF_360-15R, MGF_300-1L and 2R, MGF_360-18R, MGF_300-4L, and MGF110-12L, revealing greater domain/functional variety in these genes than previously appreciated.

### 3.4 Changes in domain copy number

**Figure 4.**
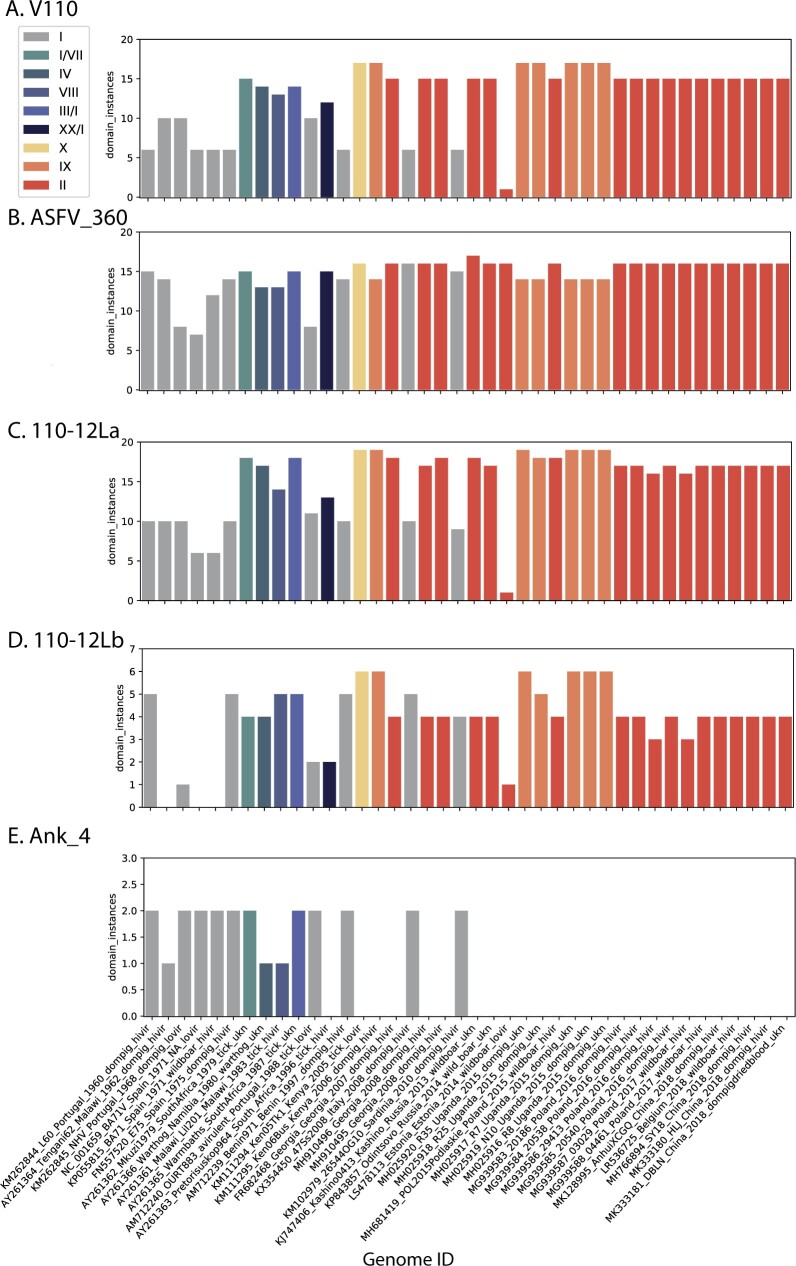
Changes in domain copy numbers. The total number of domains detected was plotted per genome, organized by sample date and colored by ASFV genotype (see legend inset for color code). Domains examined are A: Pfam v110 domain (found on MGS_110 family members), B: Pfam ASFV_360 domain (found on MGS_360 family members), C: the custom domain MGF_110-12La, D: The custom domain MGF_110-12Lb, and E: the Pfam doman Ank_4. Genome ids (*X* axis) include GenBank accession number, strain_name, country, date, host, virulence, and length in nucleotides.

MGF counts vary with ASFV genotype and also between attenuated and virulent strains. This is illustrated in Fig. 4, where we have plotted specific domain counts by sample date and virus genotype. As clearly shown in Fig. 4, viruses of genotypes GII and GIX possess higher levels of MGF_110- and MGF_360-specific domains. A few domains were observed to be absent from GII and GIX genomes, for example, an Ankyrin 4 domain found in some genotypes is not present in GII or GIX (Fig. 4).

Of potential importance to disease status, it has been observed in several analyses that changes in MGF numbers might result in altered viral properties. A deletion of a large 5′ region including multiple MGF_110 elements was associated with attenuation of an Estonian ASFV strain (Zani et al. 2018). Two GI viruses Lisboa60 (strain name L60, KM262844, a virulent strain) and NH/P68 (strain name NHV, KM262845, a nonvirulent strain) studied for their altered virulence revealed differences in four MGFs (MGF_100, MGF_110, MGF_360, and MGF_505; Portugal et al. 2015). The attenuated strain NHV showed an increase in MGF_100 and MGF-110 scores and a decrease in MGF_360 and MGF_505 scores. MGF_110-12La, an unconventional MGF_110 family member, has higher domain counts in GII strains (Fig. 4C), whereas MGF_110-12Lb, an unconventional MGF_110 family member, has the highest domain counts in GIX Uganda viruses (Fig. 4D). The Ank-4 domain is not detected in GII and GIX viruses. Ankyrin motifs are typically found in scaffolding and signaling molecules.

### 3.5 Analyses of paired viruses

Finally, we applied the genome scale domain comparison method to examine pairs of ASFV strains with reported differences in virulence. Such analyses are crucial in efforts to understand the molecular basis for attenuation or virulence and to guide efforts for vaccine design.

**Figure 5.**
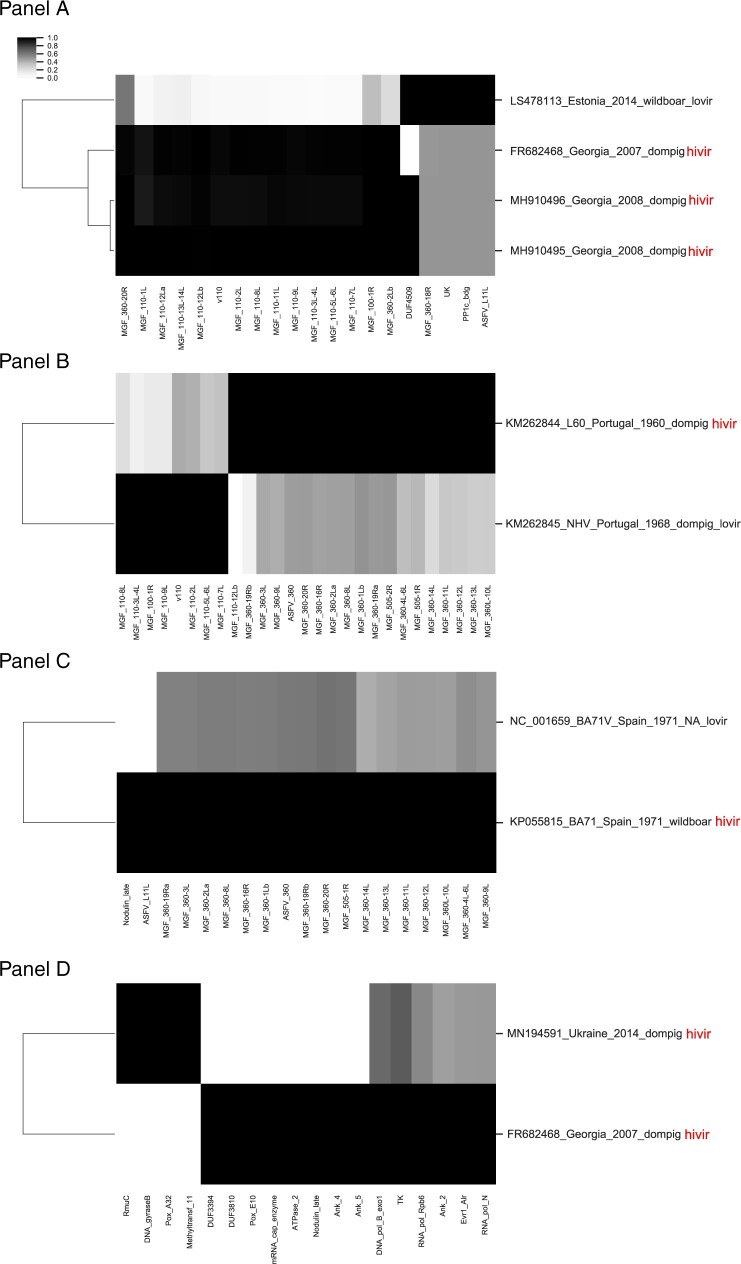
Differences in domains between paired ASFV strains. For each panel, the indicated genomes were examined for Pfam and MGF domain content, the bit-scores for all domains identified with domain_i-Evalue ≤0.0001 were collected for each domain, and a matrix was prepared and subjected to hierarchical clustering (see Section 2) based on domain whose normalized values showed ≥0.03 variance. Genome IDs (Y axis) include GenBank accession number, strain_name, country, date, host, and virulence (lovir = low-reported virulence, hivir = high-reported virulence).

For example, a naturally occurring ASFV variant was recently described from Estonia that displayed attenuation in animal tests (Zani et al. 2018). The original report noted that the Estonian variant was missing twenty-six genes including thirteen members of the MGF_110 family, three members of the MGF_360 family, deletions of MGF_100_1R, L83L, L60L, and KP177R as well as a duplication and rearrangements (Zani et al. 2018). We applied the domain classification tool to compare the variant Estonian strain to contemporary viruses from Georgia. Changes in protein domains are shown in Fig. 5A with domains showing variation across the set of four related genomes indicated by changes in the cluster map. The MGF_110 and MGF_360 changes previously noted are clearly visible with reduced signals for these two families of genes (Fig. 5A). Additional domain changes were observed including variations in the DUF4509, UK, PP1c_bdg, and ASFV_L11L domains. The DUF4509 domain is found on a subset of MGF_360 domains and is consistent with the reported MGF_360 changes. The PP1c_bdg domain is found on a Phosphatase-1 catalytic subunit binding region that may influence apoptosis (Jousse et al. 2003) and may be relevant for ASFV virulence. The ASFV_L11L domain also shows changes, and this domain is found on the L11L gene which although reported to be nonessential for virus growth (Kleiboeker et al. 1998) was previously noted to be missing from attenuated viruses (Zani et al. 2018).

Other examples include the Lisboa60 (L60) virulent strain and the NH/P68 (NHV) nonvirulent strain, which have been described and compared for virulence differences (Portugal et al. 2015). Domain differences between the two strains confirm the previously reported changes in MGFs (100, 110, 360, and 505, Fig. 5B). Also, BA71 and BA71V are a pair of virulent/attenuated ASFV strains. The BA71V strain was adapted to cell culture and showed attenuation accompanied by the loss of MGF_360 and 505 genes (Lacasta et al. 2015; Rodriguez et al. 2015). The domain differences between the two strains confirm the previously reported differences in the MGF_360 and MGF_505 genes (Rodriguez et al. 2015). In addition, the ASFV_L11L domain and a Nodulin_late domain show a change in signal in the attenuated strain (Fig. 5C). The observed changes in ASFV_L11L in two quite different pairs of virulent/avirulent ASFV strains are notable, and the role of the ASFV_L11L membrane protein should be reexamined in more detail.

## 4. Discussion

We have demonstrated the utility of a novel method of characterizing ASFV-encoded protein diversity on a genome scale based on profile HMM descriptions of conserved protein domains. The method exploits the Pfam collection of profile HMMs (Finn et al. 2014) as well as the rapid and sensitive HMMER3 software (Eddy 2011). Note our approach is neither limited to functional domains nor to the domains compiled in the extensive Pfam collection. As shown in Fig. 3, custom domains can be built and can provide additional resolution of complex genomes. The standard methods of accurately comparing large virus genomes requires the careful preparation of a full-length genome alignment of the ∼190 kb ASFV genome combined with an ML phylogenetic tree inference coupled with bootstrapping to check the reliability of the topology of the resulting phylogenetic tree. The combined phylogenetic analysis might take several days to complete and is further complicated by the large size and frequent gene deletions and duplications in the ASFV genome, making an accurate and reproducible alignment quite difficult to generate. In comparison, the domain method described here requires no genome alignment and can be performed from an unaligned fasta file of the genome sequences through to hierarchical clustering in minutes. The clustermap analyses reported for forty-seven ASFV full genomes was performed in ∼3 min run-time on a standard laptop (in this case a 2018 MacBook Pro with 2.7 GHz Intel Core i7, and 16 GB of memory). The method will be useful for quality control of newly assembled genomes and for exploring novel ASFV genomes as they are sequenced and annotated, as well as for comparing genomes with varied clinical, epidemiological, and phenotypic outcomes. The combination of our approaches with the viral outcomes are important in efforts to develop an effective and safe ASFV vaccine.

We have identified greater diversity in the five MGFs than previously noted. We further reveal the presence of a set of unconventional MGFs (Fig. 3) that appear distinct to specific strains of ASFV. Their presence and evolution will need to be monitored in future studies. Indeed, the process of MGF evolution may be an important part of ASFV evolution and the current work provides novel tools for monitoring changes in these possibly high consequence genes. Grouping MGF genes in only five categories may result in a loss of information, obscuring important details necessary for understanding ASFV transmission, virulence, and attenuation.

The domain method described here also allows a rapid assessment in both the qualitative features of encoded domains and reports a bit-score for each identified domain, which is a protein distance from the model domain. Furthermore, the method also reports copy number changes in domains. For example, examining changes in domain instances showed that the GII ASFV strains, responsible for large global outbreak of ASF, encoded a substantial increase in several MGF gene families (Fig. 4). These changes may be an important part of the replication success of the virus and warrant further investigation.

The added benefit of domain-based classification as described here is that there is no requirement to prepare an alignment of the query genomes. The resolution of any phylogenetic constructions relies heavily on accurate alignment of homologous regions of sequences. In the case of ASFV, there are differences in MGFs across different ASFV strains, either duplications or deletions, which are very difficult and time-consuming to reliably align. Furthermore, if certain genes are missing from some of the genomes for some of the alignment, this region of the alignment may be masked in the entire alignment and will not contribute to the phylogenetic signal. However, such deletions, duplications, or inversions of domains are captured by the domain scoring system used and may be an important component of the increased resolution of the domain method.

In conclusion, hierarchical clustering based on profile HMM domain scores has provided a rapid method for comparing similar genomes to identify differences in the encoded proteins. It is not intended to replace genome-scale evolutionary analysis, rather it complements standard phylogenetic approaches by focusing on shared functional information in virus genomes. We applied the method to three sets of ASFV genomes from contemporary outbreaks with known phenotypic differences in their ability to replicate in and kill pigs (Fig. 5). The novel method identified previously noted differences (primarily in the encoded MGF genes) but revealed an additional set of changes that should be further explored as potential virulence factors. These functions may be important to remove or alter in efforts to generate attenuated yet immunogenic viruses. The computational tools for performing this analysis are openly available as a platform independent Docker image of the tool and instructions for installing and using the tool have been made available.

## Data availability

The computational tools for performing this analysis can be downloaded as a platform independent Docker image using this command (docker pull matthewcotten/asfv_class_tool). Instructions for installing and using the tool are available in the Supplementary Data Readme file.

## Funding

This work was supported by a Marie Sklodowska-Curie Individual Fellowship, funded by European Union’s Horizon 2020 Research and innovation programme (M.V.T.P., Grant No. 799417), by a Wellcome Trust Intermediate Fellowship (C.M., Grant No. 105684/Z/14/Z), by the ASF-RESIST African Union Commission (C.M., D.L.R., Grant No. AURG‐II‐1‐196‐2016), and MRC (M.C., D.L.R., MC UU 1201412).

**Conflict of interest:** None declared.

## Supplementary Material

veaa044_Supplementary_Data
