# Supplementary material for: Increased resolution of African swine fever virus genome patterns based on profile HMMs of protein domains
Source: Virus Evol. 2020 Jun 19;6(2):veaa044. doi: 10.1093/ve/veaa044 (PMC7474929; doi:10.1093/ve/veaa044)
Supplement: veaa044_Supplementary_Data [file ve_6_2_veaa044_s6.zip › Supplementary_Table_1_ASFV_47_Genomes_Master.pdf]

Supplementary Table 1

	GenBank_Acc	ID	Collection_date	Host	Virulence	Genotype
1	AY261360	AY261360_Kenya_1950_Kenya_1950_dompig_hivir	1950	dompig	hivir	X
2	KM262844	KM262844_L60_Portugal_1960_dompig_hivir	1960	dompig	hivir	I
3	AY261364	AY261364_Tengani62_Malawi_1962_dompig_hivir	1962	dompig	hivir	I
4	KM262845	KM262845_NHV_Portugal_1968_dompig_lovir	1968	dompig	lovir	I
5	KP055815	KP055815_BA71_Spain_1971_wildboar_hivir	1971	wildboar	hivir	I
6	U18466	U18466_BA71V_Spain_1971_na_lovir	1971	ukn	lovir	I
7	FN557520	FN557520_E75_Spain_1975_dompig_hivir	1975	dompig	hivir	I
8	AY261362	AY261362_Mkuzi1979_SouthAfrica_1979_tick_ukn	1979	ukn	ukn	I/VII
9	AY261366	AY261366_Warthog_Namibia_1980_warthog_ukn	1980	warthog	ukn	IV
10	AY261361	AY261361_Malawi_Lil201_Malawi_1983_tick_hivir	1983	tick	hivir	VIII
11	AY261365	AY261365_Warmbaths_SouthAfrica_1987_tick_ukn	1987	tick	ukn	III/I
12	AM712240	AM712240_OURT883_avirulent_Portugal_1988_tick_lovir	1988	tick	lovir	I
13	AY261363	AY261363_Pretorisuskop964_South_Africa_1996_tick_hivir	1996	tick	hivir	XX/I
14	AM712239	AM712239_Benin971_Benin_1997_dompig_hivir	1997	dompig	hivir	I
15	KM111294	KM111294_Ken05Tk1_Kenya_2005_tick_lovir	2005	tick	lovir	X
16	KM111295	KM111295_Ken06Bus_Kenya_2006_dompig_hivir	2006	dompig	hivir	IX
17	FR682468	FR682468_Georgia_Georgia_2007_dompig_hivir	2007	dompig	hivir	II
18	KX354450	KX354450_47Ss2008_Italy_2008_dompig_hivir	2008	dompig	hivir	I
19	MH910495	MH910495_Georgia_2008_dompig_hivir	2008	dompig	hivir	II
20	MH910496	MH910496_Georgia_2008_dompig_hivir	2008	dompig	hivir	II
21	KM102979	KM102979_26544OG10_Sardinia_2010_dompig_hivir	2010	dompig	hivir	I
22	KJ747406	KJ747406_Kashino0413_Kashino_Russia_2013_wildboar_ukn	2013	wildboar	ukn	II
23	KP843857	KP843857_Odintsovo_Russia_2014_wildboar_ukn	2014	wildboar	ukn	II
24	LS478113	LS478113_Estonia_Estonia_2014_wildboar_lovir	2014	wildboar	lovir	II
25	MK628478	MK628478_Lithuania_2014_dompig_ukn	2014	dompig	ukn	II
26	MN194591	MN194591_Ukraine_2014_dompig_hivir	2014	dompig	hivir	II
27	MH025916	MH025916_R8_Uganda_2015_dompig_ukn	2015	dompig	ukn	IX
28	MH025917	MH025917_R7_Uganda_2015_dompig_ukn	2015	dompig	ukn	IX
29	MH025918	MH025918_R25_Uganda_2015_dompig_ukn	2015	dompig	ukn	IX
30	MH025919	MH025919_N10_Uganda_2015_dompig_ukn	2015	dompig	ukn	IX
31	MH025920	MH025920_R35_Uganda_2015_dompig_ukn	2015	dompig	ukn	IX
32	MH681419	MH681419_POL2015Podlaskie_Poland_2015_wildboar_hivir	2015	wildboar	hivir	II
33	MG939583	MG939583_20186_Poland_2016_dompig_hivir	2016	dompig	hivir	II
34	MG939584	MG939584_20538_Poland_2016_dompig_hivir	2016	dompig	hivir	II
35	MG939585	MG939585_20540_Poland_2016_dompig_hivir	2016	dompig	hivir	II
36	MG939586	MG939586_29413_Poland_2016_dompig_hivir	2016	dompig	hivir	II
37	MG939587	MG939587_03029_Poland_2017_wildboar_hivir	2017	wildboar	hivir	II
38	MG939588	MG939588_04461_Poland_2017_wildboar_hivir	2017	wildboar	hivir	II
39	MG939589	MG939589_05838 _Poland_2017_wildboar_hivir	2017	wildboar	hivir	II
40	LR722599	LR722599_Moldova_2017_ukn_ukn	2017	ukn	ukn	II
41	LR722600	LR722600_CzechRep_2017_ukn_ukn	2017	ukn	ukn	II
42	MH766894	MH766894_SY18_China_2018_dompig_hivir	2018	dompig	hivir	II
43	MK128995	MK128995_AnhuiXCGQ_China_2018_dompig_hivir	2018	dompig	hivir	II
44	LR536725	LR536725_Belgium_2018_wildboar_hivir	2018	wildboar	hivir	II
45	MK333180	MK333180_HLJ_China_2018_dompig_hivir	2018	dompig	hivir	II
46	MK543947	MK543947_Belgium_2018_wildboar_ukn	2018	wildboar	ukn	II
47	MK645909	MK645909_China_2018_wildboar_ukn	2018	wildboar	ukn	II
