# Supplementary figures and images for: Increased resolution of African swine fever virus genome patterns based on profile HMMs of protein domains
Source: Virus Evol. 2020 Jun 19;6(2):veaa044. doi: 10.1093/ve/veaa044 (PMC7474929; doi:10.1093/ve/veaa044)

### Consensus

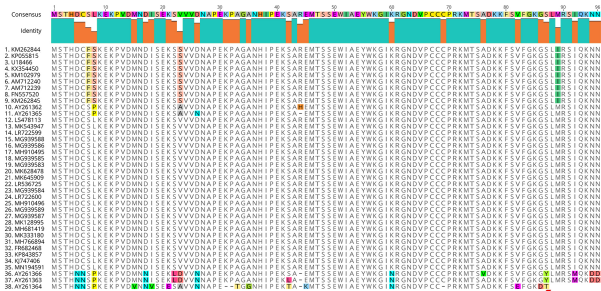

B.

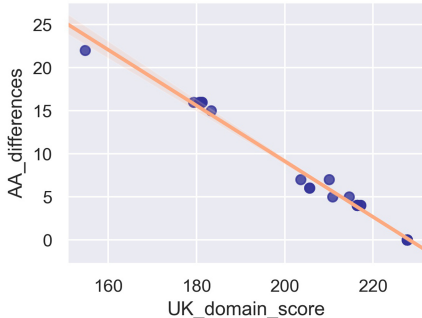

## Supplementary Figure 1

Supplement: veaa044_Supplementary_Data [file ve_6_2_veaa044_s6.zip › Supplementary_Figure_1_UK_domain.pdf]
